# Changes in clinical characteristics and outcomes of patients hospitalized with COVID-19 during two years of the pandemic: experience in a venezuelan hospital

**DOI:** 10.17843/rpmesp.2022.393.11195

**Published:** 2022-09-30

**Authors:** María C. Arvelo, María Montes de Oca, Laura Sánchez-Traslaviña, Flor H. Pujol, Rossana C. Jaspe, Isabel C. Silva, Irene Stulin, Gabriela Blanco, Jennireth Quevedo, Nathalia Valera, Irene Papa, Santiago Bacci, Fátima de Abreu, Héctor Villarroel, Juan C. Catari, José L. Lopez, Brigitte Moran, Claudio Cárdenas, Saverio Santucci, José L. Viloria, Eleonora García, Jerry Gómez, Antonio Martinelli, Manuel Guzmán

**Affiliations:** 1 Hospital Centro Médico de Caracas, Caracas, Venezuela. Hospital Centro Médico de Caracas Caracas Venezuela; 2 Universidad Central de Venezuela (UCV), Faculty of Medicine, Venezuela. Universidad Central de Venezuela Universidad Central de Venezuela (UCV) Faculty of Medicine Caracas Venezuela; 3 Laboratory of Molecular Virology, Center for Microbiology and Cell Biology. (CMBC), Instituto Venezolano de Investigaciones Científicas (IVIC), Caracas, Venezuela. Laboratory of Molecular Virology Center for Microbiology and Cell Biology. (CMBC) Instituto Venezolano de Investigaciones Científicas (IVIC) Caracas Venezuela

**Keywords:** COVID-19, SARS-CoV-2, SARS-CoV-2 variants, Intensive Care Units, Mortality, In-Hospital

## Abstract

**Objectives.:**

To determine changes in the clinical characteristics and in-hospital outcomes of patients hospitalized for COVID-19 in a private hospital in Caracas during two years of the pandemic.

**Materials and Methods.:**

Retrospective, observational study of patients hospitalized for COVID-19. We evaluated the correspondence between waves of hospital admissions and circulating variants of SARS-CoV-2 in the general population of the Capital District and Miranda state.

**Results.:**

A total of 1025 patients (569 men and 456 women) were included, with a mean age of 62.9 SD: 16.2 years. Four waves of hospital admissions were identified: first (March-November 2020) 150/1025 (14.6%) cases; second (December 2020 to May 2021) 415/1025 (40.5%) cases; third (June-December 2021) 344/1025 (33.6%) cases; fourth (January-February 2022) 116/1025 (11.3%) cases. The mean age was higher in the fourth wave (first: 64.0±15.7, second: 61.4±15.8, third: 62.1±16.5, and fourth wave: 68.5±16.4), while the proportion of male patients (first: 66.7%, second: 58.8%, third: 50.3%, and fourth wave: 44.8%), patients with severe-critical illness (first: 65.3%, second: 57%, third: 51.7%, and fourth wave: 44.8%), in-hospital stay (first: 9.1±6.0, second: 9.0±7.3, third: 8.8±7.7, and fourth wave: 6.9±5.0 days), ICU admissions (first: 23.3%, second: 15.7%, third: 14.0%, and fourth wave: 11.2%; p=0.027) and mortality (first: 21. 8%, second: 10.7%, third: 9.1%, and fourth wave: 7.1%; p<0.001) progressively decreased over time.

**Conclusions.:**

The results show lower frequency of severe cases and improvement of in-hospital outcomes in two years of the pandemic. Changes in circulating variants, improvements in disease management and vaccination are likely to have influenced these results.

## INTRODUCTION

Coronavirus disease 2019 (COVID-19) is the first pandemic in more than 100 years. In Venezuela, according to data from CENDES-COVID-19, 515,126 cumulative cases (81,829 cases in Distrito Capital and 71,781 in Miranda state) and 5636 deaths (1074 deaths in Distrito Capital and 624 in Miranda state) were reported to date ^1^.

More than 2000 SARS-CoV-2 lineages have been described so far, some of which have been designated by the World Health Organization (WHO) as variants of interest (VOI), or variants of concern (VOC), given their impact on public health. WHO identified five VOCs (alpha, beta, gamma, delta and omicron) since the beginning of the pandemic and several VOIs, including lambda and mu, which were identified in Latin America. By the end of March 2022, the omicron VOC predominated worldwide ^2^.

Different strategies have been implemented to reduce the transmission of the virus and its impact on health since the beginning of the pandemic. Restrictive measures, such as the use of masks, social distancing and mass closures, reduced the risk of infection ^3^ ^-^ ^5^. Several therapeutic interventions have been implemented in hospitals, such as the use of antivirals, anti-inflammatory drugs, thrombosis prophylaxis and oxygen therapy. Finally, the development of vaccines since 2021 has led to a reduction of COVID-19 cases ^6^ ^-^ ^8^.

Several studies have evaluated the clinical characteristics and outcomes of patients hospitalized due to COVID-19 in different moments of the pandemic ^9^ ^-^ ^19^. Although in some studies the number of hospitalized patients was relatively similar ^9^ ^,^ ^11^ ^,^ ^13^ ^,^ ^14^ ^,^ ^18^ between waves, disease severity, intensive care unit (ICU) admissions, and mortality tended to decrease over time ^9^ ^-^ ^19^.

We did not find any previous study that analyzed local temporal trends in the clinical characteristics, severity, in-hospital course, and mortality of patients hospitalized due to COVID-19 in Venezuela. This information may help to better understand the burden of COVID-19 in our local hospital system and contribute to the definition of public policies at the regional level. In order to address this gap, this study was carried out in patients hospitalized due to COVID-19 in the private hospital Centro Médico de Caracas (CMC) with the aim of determining changes in the profile of patients and their in-hospital outcomes during two years of pandemic.

**KEY MESSAGES**

Motivation for the study: no study has analyzed locally the temporal trends in the characteristics of patients hospitalized due to COVID-19, their in-hospital course and mortality.

Main findings: we found that 1025 patients were admitted to a hospital in Caracas between March 2020 and February 2022 (14.6% in the first wave, 40.5% in the second, 33.6% in the third and 11.3% in the fourth). The percentage of male patients, with severe-critical illness, in-hospital stay, ICU admissions and mortality decreased over time.

Implications: improvements are probably associated with circulating variants of SARS-CoV-2, improving clinical management and the vaccination program.

## MATERIALS AND METHODS

A retrospective cohort study (review of medical records) of patients hospitalized and diagnosed with COVID-19 at the CMC hospital between March 1, 2020 and February 28, 2022. The CMC hospital is a private hospital in the metropolitan area of Caracas that has all the medical and assistance resources of a Type A private hospital, according to the COVENIN 2339:87 Standard ^20^.

Four waves were identified during the two years of the pandemic based on the number of hospitalized cases at CMC. The first wave occurred between March and November 2020 (9 months), the second wave occurred between December 2020 and May 2021 (6 months), the third wave occurred between June and December 2021 (7 months) and the fourth and final wave was between January and February 2022 (2 months).

The case report form (CORE Spanish version dated April 23, 2020) developed by the ISARIC study and WHO ^21^ was used to collect patient data. Data collected included age (years), stage group, sex, diagnostic test for COVID-19 (antigen-SARS-CoV-2, rRT-PCR, and IgM-IgG), number of days with symptoms prior admission, admission oxygen saturation at room air (SpO_2_ %), severity of illness (mild, moderate, severe-critical), treatments (antiviral, antibiotic and anti-inflammatory), days of hospitalization, days in ICU, requirement for invasive mechanical ventilation, laboratory tests and admission chest CT scans.

### COVID-19 Diagnostic Procedures

The diagnosis of COVID-19 was based on SARS-CoV-2 antigen detection or the real-time reverse transcription-polymerase chain reaction (rRT-PCR) test result for SARS-CoV-2. Respiratory tract samples (nasopharyngeal swabs) were collected according to WHO guidelines ^22^.

### Criteria for hospitalization and classification of severity of COVID-19

The most common criterion for hospital admission was hypoxemia on room air and/or presence of pulmonary infiltrates. Admission to the ICU was reserved for patients with severe acute respiratory failure requiring oxygen therapy with high-flow nasal cannula or invasive mechanical ventilation (IMV). Disease severity was established according to National Institute of Health criteria ^23^.

### Semiquantitative analysis of chest CT

Pulmonary involvement and definitions of radiological terms (ground glass, consolidated, cobblestone pattern) were based on the Fleischner Society glossary ^24^. The total admission chest CT severity score was calculated according to the following criteria of Pan *et al*. (25): 0 (no involvement), 1 (involvement <5%), 2 (involvement 5 to 25%), 3 (involvement 26 to 50%), 4 (involvement 51% to 75%), 5 (involvement >75%). The total score was the sum of the involvement of each one (range 0 to 25).

In order to distribute the patients into severity quartiles of the total score on chest CT, we used the cut-off points of the first, second and third quartiles by entering the score of all patients in a statistical quartile calculator. The cutoff points for the quartiles were: Q1 (score between 0-8 points), Q2 (score between 9-12 points), Q3 (score between 13-16 points) and Q4 (score between 17-25 points) ^(26)^. Each patient was assigned to the corresponding quartile (e.g., a CT score of 14 points places the patient in Q3).

### Genomic analysis of SARS-CoV-2 variants

SARS-CoV-2 variants were identified in samples from the general population of the Capital District and Miranda state, based on sequencing of a small fragment of the viral genome surface gene, with confirmation of variant identification by whole genome sequencing ^27^ ^-^ ^30^.

No variants are expected to have circulated during the first wave, most lineages probably belonged to group B, which possess the D614G mutation in the spike protein ^27^. The second wave was characterized by the circulation of the gamma variant along with other nonvariant lineages during January and February 2021, which were totally displaced by gamma OCV from March 2021 (>90%) ^28^. The third wave was characterized by a greater diversity of variant circulation, both VOI and VOC, in which VOC gamma predominated in June 2021. In August 2021, there was a similar circulation frequency of VOC gamma and delta, and of VOI mu in smaller proportion. Then delta was the most abundant from September 2021 until the end of the year. The fourth wave was characterized by the almost exclusive circulation of omicron VOC ^30^.

### Statistical analysis

Descriptive statistics show categorical variables as frequency in number and percentage, while quantitative variables are shown as median and interquartile range (Q75-Q25). Comparison of quantitative variables among patients in the four waves was carried out by using the nonparametric Kruskal Wallis test because the variables did not show normal distribution (Supplementary Table S1). For qualitative variables we used Pearson’s chi-square test. A p-value of 0.05 or less was considered statistically significant. STATISTICA Version 10 (StatSoft) was used for the analyses.

### Ethical Aspects

The study was approved by the Bioethics Commission of the C.A. Centro Médico de Caracas. Since this was a retrospective study involving the review of medical records, the confidentiality of the patients was preserved by the researchers.

## RESULTS

A total of 1025 adult patients (569 males and 456 females) were admitted with diagnosis of COVID-19 between March 1, 2020 and February 28, 2022. The mean age was 62.9, SD: 16.2 years. Sixteen patients were transferred to other institutions and one patient remained hospitalized by the end of the study, so mortality details were analyzed for 1008 patients.

**Figure 1:**
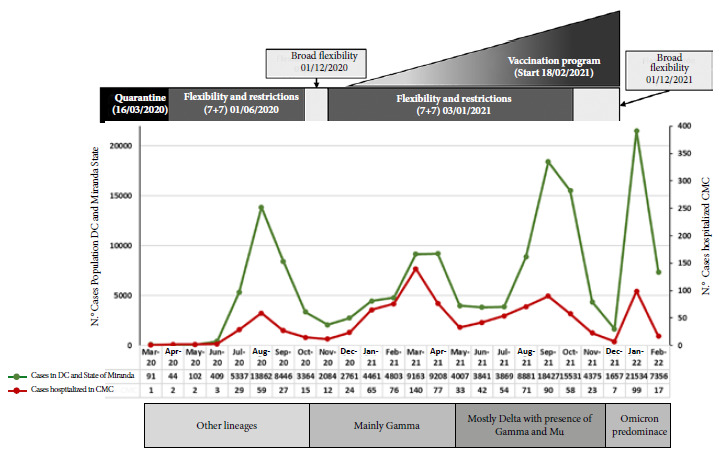
Number of cases detected with COVID-19 in the general population (Capital District and Miranda State) and number of patients hospitalized due to COVID-19 according to month of admission at the Caracas Medical Center.

Figure 1 shows the four waves according to the number of patients hospitalized due to COVID-19 per month of admission and the number of cases per month registered by CENDES in the general population of the states of Distrito Capital and Miranda ^1^. In both populations, we identified four waves that coincide in time. Regarding hospitalized patients, 150/1025 (14.6%) cases were recorded in the first wave (peak in August; 59 cases), 415/1025 (40.5%) cases in the second wave (peak in March 2021; 140 cases), 344/1025 (33.6%) cases in the third wave (peak in September; 90 cases), and 116/1025 (11.3%) cases in the fourth wave (of rapid ascent and short duration) (peak in January; 99 cases). Similar characteristics were found in the case curve of the general population. The circulating variants during each wave in the Capital District and Miranda state (entities where most of the hospitalized patients come from) are shown in Figure 1. The clinical characteristics, severity of the disease, in-hospital stay, measurement used of the total number of patients and in each wave are shown in Table 1. The predominant test for diagnosis during the first and second waves (100 and 67%, respectively) was rRT-PCR, while antigen detection (SARS-CoV-2) was the most frequent during the third and fourth waves (66 and 60%, respectively). Patients during the fourth wave were older. The proportion of male patients, severe-critical illness, days of symptoms prior to admission and in-hospital stay decreased significantly with the progression of the pandemic. Most patients during the first and second waves were males (67 and 59%, respectively) and had severe-critical illness (65 and 57%, respectively), while those in the fourth wave were mostly females (55%) and had a lower proportion of severe-critical patients (45%). SpO_2_ on admission was significantly lower in patients in the first wave (p = 0.030). A total of 87% of patients received remdesivir, 34.1% antibiotics, 70.3% systemic steroids and 3.6% tocilizumab. Only 41.3% of patients during the first wave received remdesivir compared with more than 90% of those in the second, third and fourth waves. Antibiotic use was over 55% in the first wave compared to approximately 30% during the other waves. There was a progressive decrease in the use of systemic steroids as the pandemic progressed and increased use of tocilizumab in the third and fourth waves.

**Table 1:** Clinical characteristics, hospital stay, medication use and invasive mechanical ventilation of patients hospitalized due to COVID-19 in the four waves.

Variables	Total n=1025 n (%)	Wave 1 n=150 n (%)	Ola 2 n=415 n (%)	Wave 3 n=344 n (%)	Wave 4 n=116 n (%)	p-value
Age	63.5 (23.0)	66.0 (22.0)	62.0 (22.0)	62.0 (25.0)	72.5 (21.5)	<0.001^a^
Age group, (%)						
≤30 years	28/1023 (2.8)	3/150 (2.0)	11/415 (2.7)	11/344 (3.2)	3/116 (2.6)	0.043^b^
31-50 years	210/1023 (20.5)	30/150 (20.0)	93/415 (22.4)	75/344 (21.8)	12/116 (10.3)	
51-65 years	297/1023 (29.0)	36/150 (24.0)	128/415 (30.8)	100/344 (29.1)	30/116 (25.9)	
>65 years	488/1023 (47.7)	81/150 (54.0)	183/415 (44.1)	158/344 (45.9)	71/116 (61.2)	
Sex, (%)						
Male	569/1025 (55.5)	100/150 (66.7)	244/415 (58.8)	173/344 (50.3)	52/116 (44.8)	<0.001^b^
Female	456/1025 (44.5)	50/150 (33.3)	171/415 (41.2)	171/344 (49.7)	64/116 (55.2)	
COVID-19 Diagnosis, (%)						
Antigen (SARS-CoV-2)	415/985 (42.1)	0/143 (0)	126/392 (32.1)	219/334 (65.6)	70/116 (60.3)	<0.001^b^
rRT-PCR	567/985 (57.6)	143/143 (100)	264/392 (67.4)	114/334 (34.1)	46/116 (39.7)	
IgM-IgG	3/985 (0.3)	0/143 (0.0)	2/392 (0.5)	1/334 (0.3)	0/116 (0)	
Days of symptoms prior to admission	7.0 (5.0)	7.0 (5.0)	7.0 (5.0)	7.0 (5.0)	6,0 (4.0)	<0.001^a^
Stages days with symptoms, (%)						
Early (0-5 days)	360/975 (36.9)	52/149 (34.9)	124/384 (32.3)	129/328 (39.3)	55/114 (48.2)	0.032^b^
Progressive (6-8 days)	331/975 (33.9)	46/149 (30.9)	135/384 (35.2)	113/328 (34.4)	37/114 (32.5)	
Peak (9-13 days)	188/975 (19.3)	31/149 (20.8)	89/384 (23.2)	53/328 (16.2)	15/114 (13.2)	
Late (≥14 days)	96/975 (9.9)	20/149 (13.4)	36/384 (9.4)	33/328 (10.1)	7/114 (6.1)	
SpO_2_ % Admission, (FiO_2_ 0,21)	94.0 (6.0)	93.0 (7.0)	94.0 (6.0)	95.0 (6.0)	95.0 (6.0)	0.030^a^
Severity COVID-19, (%)						
Mild	68/1024 (6.6)	7/150 (4.7)	12/414 (2.9)	22/344 (6.4)	27/116 (23.3)	
Moderate	392/1024 (38.3)	45/150 (30.0)	166/414 (40.1)	144/344 (41.9)	37/116 (31.9)	<0.001^b^
Severe-Critical	564/1024 (55.1)	98/150 (65.3)	236/414 (57.0)	178/344 (51.7)	52/116 (44.8)	
Remdesivir, (%)	896/1025 (87.4)	62/150 (41.3)	405/415 (97.6)	324/344 (94.2)	105/116 (90.5)	0.027^b^
Antibiotics, (%)	349/1024 (34.1)	85/150 (56.7)	125/415 (30.1)	104/344 (30.2)	35/115 (30.4)	<0.001^b^
Systemic steroids, (%)	721/1025 (70.3)	132/150 (88)	312/415 (75.2)	211/344 (61.3)	66/116 (56.9)	<0.001^b^
Tocilizumab, (%)	37/1025 (3.6)	0/150 (0)	2/415 (0.5)	26/344 (7.6)	9/116 (7.8)	<0.001^b^
Hospitalization days	6.0 (4.0)	8.0 (7.0)	7.0 (3.0)	6.0 (3.0)	6.0 (3.0)	<0.001^a^
Days in ICU	10.0 (10.0)	9,5 (9.5)	10.0 (11.0)	12,5 (15.5)	6.0 (4.0)	0.036^a^
IMV, (%)	150/1024 (14.6)	26/150 (17.3)	47/344 (11.3)	40/344 (11.6)	12/116 (10.3)	0.217^b^

rRT-PCR: real-time reverse transcription-polymerase chain reaction test; IgM-IgG: immunoglobulin G-M; SpO_2_: pulse oximetry oxygen saturation; ICU: intensive care unit; IMV: invasive mechanical ventilation, (%).

Data are shown as n (%) or median (interquartile range).

a Kruskal Wallis test, ^b^ Pearson’s chi-square test.

Table 2 shows the values of some laboratory tests at admission for the total of patients and all the waves. Patients during the first wave showed significantly higher values for leukocyte count, neutrophils, platelets, and BUN, while those in the fourth wave had lower levels of hemoglobin, hematocrit, ferritin, and vitamin D. No differences were found in the values of lymphocytes, glycemia, CRP, and HDL.

**Table 2:** Admission laboratory results of the total number of patients hospitalized due to COVID-19 during the four waves.

Variables	Total n=1025	Wave 1 n=150	Wave 2 n=415	Wave 3 n=344	Wave 4 n=116	p-value ^a^
Hemoglobin, gr/dL	14.1 (2.3)	14.4 (2.2)	14.5 (2.2)	13.5 (2.0)	13.2 (1.9)	<0.001
Hematocrit, (%)	42.6 (7.0)	43.3 (6.7)	44.9 (6.6)	41.0 (6.5)	40.4 (5.7)	<0.001
Leucocytes, (x10^9^/L)	6700 (4400)	7300 (4900)	6600 (4450)	6300 (3800)	7300 (4600)	0.001
Neutrophiles, (x10^9^/L)	4900 (4200)	5600 (4151)	4900 (4200)	4600 (3800)	5000 (4450)	0.005
Lymphocytes, (x10^9^/L)	900 (700)	981.5 (700)	900 (700)	900 (700)	1000 (650)	0.168
Platelets, (x10^9^/L)	203.0 (106.0)	224.0 (132.0)	201.0 (97.5)	193.0 (97.0)	216.0 (93.0)	0.004
CRP, (mg/dL)	5.05 (8.4)	5.6 (6.0)	4.8 (9.3)	4.8 (8.8)	4.8 (9.9)	0.507
Baseline LDH, (u/L)	273.0 (137.5)	286.5 (165.5)	276.5 (142.0)	271.0 (137.0)	258.0 (95.0)	0.223
D dimer, (µg/L)	0.83 (1.07)	1.02 (2.01)	0.68 (0.73)	0.80 (0.88)	1.49 (2.12)	<0.001
Ferritin, (ng/mL)	395.1 (464.8)	577.1 (483.7)	384.4 (439.5)	443.1 (581.0)	217.0 (303.5)	<0.001
Glucose, (mg/dL)	108 (38)	107 (40)	106 (37)	109 (35)	113 (46)	0.125
Creatinine, (mg/dL)	1.0 (0.33)	0.99 (0.29)	0.97 (0.33)	1.02 (0.36)	1.01 (0.39)	0.018
BUN, (mg/dL)	15.0 (8.0)	17.4 (13.6)	15.0 (8.2)	14.0 (6.0)	16.0 (7.0)	<0.001
25 (OH) Vitamin D, (ng/mL)	25.8 (16.9)	24.7 (14.7)	26.2 (16.0)	29.1 (19.5)	16.6 (16.9)	<0.001

CRP: C-reactive protein, LDH: lactate dehydrogenase, BUN: urea nitrogen.

Data are shown as median (interquartile range).

a Kruskal Wallis test

**Figure 2:**
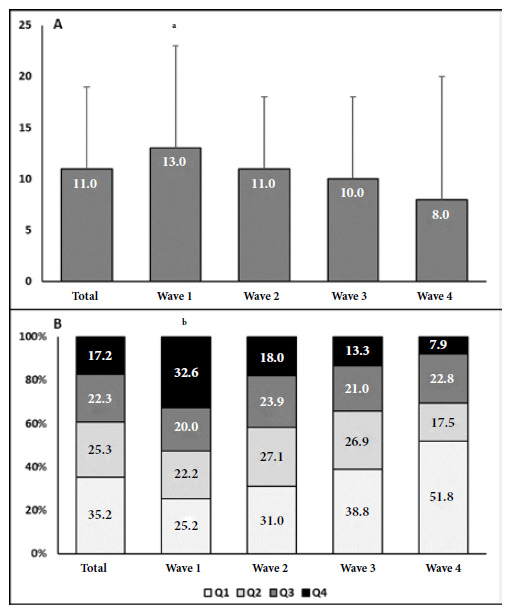
A. Chest CT severity score in the total patient population and in the four waves. B. Distribution of patients in chest CT severity quartiles in the total population and in the four waves.

The severity on admission chest CT score and the distribution of patients according to the quartile of tomographic severity that patients presented at admission of the total patients and the four waves are shown in Figures 2A-2B. The severity score progressively decreased with pandemic time in the most severe quartiles (Q3-Q4). The highest score was observed during the first wave and the lowest in the fourth wave. Some 52.6% of patients in the first wave were in the most severe quartiles (Q3-Q4), compared with 31.3% of those in the Q3-Q4 quartiles in the fourth wave.

**Figure 3:**
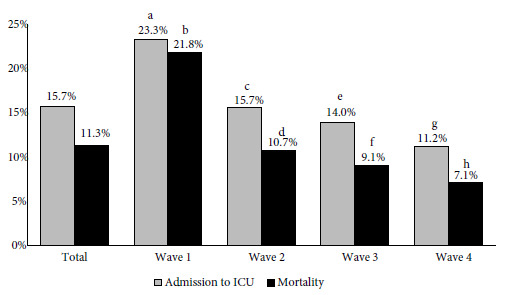
Proportion of patients hospitalized due to COVID-19 with intensive care unit (ICU) admissions and total mortality, in the four waves.

**Figure 4:**
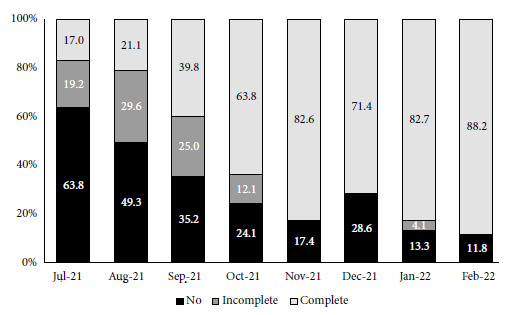
COVID-19 vaccination schedule for patients hospitalized between July and December 2021.

ICU admissions and mortality for total patients as well as in the waves are shown in Figure 3. A total of 161/1025 (15.7%) patients were admitted to the ICU and the overall mortality was 11.3% (114/1008). As the pandemic progressed, we observed progressive decreases in ICU admissions (first 23.3%, second 15.7%, third 14.0%, and fourth 11.2%; p = 0.027) and mortality (first 21.8%, second 10.7%, third 9.1%, and fourth 7.1%; p < 0.001). When comparing ICU admissions and mortality between waves, there was only a significant difference between the first wave and the second, third, and fourth waves (Figure 4).

Figure 4 shows the COVID-19 vaccination scheme applied to patients admitted between July 2021 and February 2022. There was a progressive decrease in the proportion of unvaccinated patients and an increase in those with a complete scheme over time. In the third wave, 39.4% of patients had a complete vaccination scheme compared to 83.5% of those in the fourth wave. Only 2/178 patients (1.1%) in the third wave and 20/100 patients (20%) in the fourth wave had booster doses. Of the 39 patients who died in the third and fourth waves, 16 had a complete vaccination scheme. The vaccines most commonly used as primary scheme were: Sinopharm or Sinovac with 178 cases (64.3%), Sputnik V with 88 cases (31.7%), Pfizer with nine cases (3.2%), Moderna with one case (0.4%) and Johnson & Johnson with one case (0.4%).

## DISCUSSION

The main findings of this study regarding clinical characteristics and outcomes of patients hospitalized for COVID-19 in two pandemic years were the following. We observed differences in the number of cases hospitalized during the pandemic. In the first wave, 14.6% of the total number of patients were admitted, while in the fourth wave, 11.3% were admitted. The second and third waves had the highest number of admissions (40.5% and 33.7%, respectively). A similar behavior was observed in the number of cases registered in the general population. We also found that the mean age was higher during the fourth wave, while the proportion of male patients, severity of illness, in-hospital stay, ICU admissions, and mortality decreased progressively over time.

Several studies have evaluated the change in the number of hospital admissions for COVID-19 in the first waves of the pandemic ^9^ ^-^ ^11^ ^,^ ^13^ ^,^ ^14^ ^,^ ^17^ ^-^ ^19^. In the United States a 2020 analysis reported 11,901 patients admitted between March and April, 4116 between May and June, 2709 between July and August, and 2010 between September and November ^10^. European studies report an increase in hospital admissions for COVID-19 during two epidemic periods in 2020 ^9^ ^-^ ^11^ ^,^ ^13^ ^,^ ^14^ ^,^ ^17^ ^-^ ^19^. A study in Spain reported 2547 patients hospitalized during the first wave (February and May 2020) and 2673 during the second wave (June and September 2020) ^13^. Data from a hospital in Madrid indicate 1788 admissions (48.6%) between March and June 2020, 926 (25.2%) between July and November 2020, and 962 (26.2%) between December 2020 and April 2021. Similar trends were reported in Germany and Italy^ (^ ^11^ ^,^ ^17^ ^,^ ^19^. A study in Brazil reported 325,903 (48.05%) patients hospitalized during the first wave (February and November 2020) and 352,332 (51.95%) during the second wave (November 2020 and April 2021) ^18^.

Although the trend in the number of hospitalizations for COVID-19 in our study is in line with previous studies, showing a progressive increase in admissions between the first and second waves with a subsequent decrease in the third and fourth waves, it is important to note that the results are difficult to compare, since the waves between the studies are unequal because they take place in regions with different epidemiological curves that have probably been influenced by seasonal differences in the circulating variants, restrictive measures, and vaccination. It is likely that the main factors that have influenced the high number of admissions during the second and third waves in our study are the high transmissibility and pathogenicity of the circulating variants (gamma and delta) and the relaxation of restrictive measures. On the other hand, the predominance of the omicron variant (of high transmissibility and low pathogenicity) during the fourth wave, plus the progress in vaccination, are probably the factors with the greatest influence on the behavior of admissions during that period ^30^ ^-^ ^32^.

Studies comparing the waves in a pandemic year generally show a predominance of male patients ^9^ ^,^ ^10^ ^,^ ^13^ ^,^ ^17^ ^-^ ^19^, although some highlight a slight increase in women over time ^10^. They also show older age and worse severity parameters in the first waves ^9^ ^,^ ^10^ ^,^ ^13^ ^,^ ^17^ ^,^ ^18^. On the other hand, in-hospital outcomes showed a tendency to improve over time in other series ^9^ ^-^ ^11^ ^,^ ^13^ ^,^ ^14^ ^,^ ^17^. Some studies reported a decrease in in-hospital stay (22 vs. 14 days), ICU admissions (17.1% vs. 7.2%) and mortality (24.0% vs. 13.2%) between the first and second wave ^9^. Data in the United States from 2020 indicate that mortality was: 19.1% between March and April, 11.9% between May and June, 11.0% between July and August, and 10.8% between September and November ^10^. A study in Spain showed that ICU admissions were 16% and 10%, and cumulative mortality were 38% and 32% in the first and second waves, respectively ^13^. Similar results on mortality have been reported in Italy ^17^. Other authors have reported that hospital stay (14.5 vs. 8 days) and ICU admissions (31.9% vs. 13.3%) decreased between waves, but not mortality (14.1 vs. 11.4%) ^14^. In contrast, other studies found no differences in ICU admissions or mortality or report an increase in these outcomes ^18^ ^,^ ^19^.

There is little information on the clinical characteristics and in-hospital outcomes of patients with COVID-19 beyond the first year of the pandemic. Recent studies report decreased severity of illness during the omicron wave compared with earlier periods ^32^ ^-^ ^35^. In California, patients hospitalized during the omicron period were found to be older (66 vs. 60 years), less severe, had fewer ICU admissions (16.8 % vs. 23.3 %), less need for IMV (9.2 % vs. 13.6 %), and fewer deaths (4.0 % vs. 8.3 %) compared with those during the delta period ^34^. Part of this effect seems to be due to a higher proportion of vaccinated patients ^34^.

The findings of our study are in line with previous reports, showing a predominance of male patients in the initial waves and progressive improvement of the clinical and tomographic parameters of the disease, as well as a reduction in hospital stay, ICU admissions and mortality. Likewise, during the period of predominance of the omicron variant, we also found an increase in age, a higher proportion of women, less severity in chest CT, a decrease of hospital stay, ICU admissions, and mortality.

Multiple factors may help to justify our findings. One explanation for the differences in mortality could be the improvements in clinical management of patients including pharmacotherapy such as the use of anticoagulants, and systemic steroids. Other changes in more severe patients such as more rational use of antibiotics or tocilizumab are probably associated with the results. The decrease in age (10% lower in the >65 years group) and in the proportion of men (8-16% lower) in the second and third waves compared to the first may also have influenced the decrease in case fatality. It is likely that the increase in hospitalization of younger groups during the second and third waves may be partially related to vaccination, which initially prioritized older age groups. Finally, the decrease in severity and improvement of outcomes could also be partially explained by changes in circulating variants ^27^ ^-^ ^30^. During the first wave no variant circulated, but the second wave was characterized by the circulation of the gamma variant, the third by the gamma, delta and mu variants, and the fourth by omicron ^27^ ^-^ ^30^. Although mutations in the new variants are associated with higher infectivity and viral load, the evidence indicates an association with lower disease severity. During the period of predominance of omicron, in addition to the lower pathogenicity of this variant, it is likely that the development of vaccination (complete schemes > 80% of patients), even with booster doses, is associated with lower severity of cases, decreased admission to the ICU and mortality ^34^. One of our findings that is consistent with the lower pathogenicity of omicron and the protection of the vaccines would be the better outcomes (ICU admissions and mortality) observed in this period despite patients being older (61% ≥ 65 years).

This study has some limitations. First, it is a retrospective observational study, so there are unmeasured confounders that lack the level of detail necessary for an extensive analysis of outcomes. Nevertheless, these results are consistent with those reported in other series and represent the first analysis in a large sample of patients hospitalized for COVID-19 in our population during two years of pandemic, with information on locally circulating variants. Second, the study takes place in a private hospital in Caracas, which may influence patient characteristics and limit the generalizability of the results to other settings. Third, the study only analyzed hospitalized patients who, overall, had moderate to severe-critical disease, which limits the generalization of the findings to less severe patients treated on an outpatient basis. Fourth, we did not analyze the infecting variant in each of the hospitalized patients, but rather assessed the correspondence of each wave with the circulating viral diversity in each period.

In conclusion, the results of this study show changes over time in the clinical characteristics of patients hospitalized with COVID-19 and progressive improvement in disease severity and in-hospital outcomes. Changes in circulating variants, improved disease management, and vaccination are likely to have influenced outcomes. The findings may help to better understand the evolution of patients with COVID-19 requiring in-hospital management in our setting and contribute to the definition of health policies.
