# Supplementary material for: Changes in clinical characteristics and outcomes of patients hospitalized with COVID-19 during two years of the pandemic: experience in a venezuelan hospital
Source: Rev Peru Med Exp Salud Publica. 2022 Sep 30;39(3):292–301. doi: 10.17843/rpmesp.2022.393.11195 (PMC11397598; doi:10.17843/rpmesp.2022.393.11195)
Supplement: Supplementary files. — supplementary material. [file rpmesp-39-03-11195-s001.docx]

**Tabla S1. Prueba de normalidad de las variables cuantitativas (Kolmogorov-Smirnov con corrección de Lilliefors)**

| **Variable** | **K – S (d)** | **K – S (p)** | **Lilliefors (p)** |
| --- | --- | --- | --- |
| **Edad, años** | 0.04804 | < 0.05 | < 0.01 |
| **SpO_2_ % ingreso, (FiO2 0.21)** | 0.18070 | < 0.01 | < 0.01 |
| **Días síntomas previo ingreso** | 0.14259 | < 0.01 | < 0.01 |
| **Días hospitalización** | 0.25724 | < 0.01 | < 0.01 |
| **Días en UCI** | 0.21300 | < 0.01 | < 0.01 |
| **Hemoglobina, gr/dl** | 0.04276 | < 0.10 | < 0.01 |
| **Hematocrito, (%)** | 0.03750 | < 0.15 | < 0.01 |
| **Leucocitos, (x10^ 9/l)** | 0.13776 | < 0.01 | < 0.01 |
| **Neutrófilos, (x10^ 9/l)** | 0.14687 | < 0.01 | < 0.01 |
| **Linfocitos, (x10^ 9/l)** | 0.14253 | < 0.01 | < 0.01 |
| **Plaquetas, (x10^ 9/l)** | 0.09105 | < 0.01 | < 0.01 |
| **PCR, (mg/dl)** | 0.15010 | < 0.01 | < 0.01 |
| **LDH basal, (u/l)** | 0.15119 | < 0.01 | < 0.01 |
| **Dímero D, (µg/l)** | 0.29558 | < 0.01 | < 0.01 |
| **Ferritina, (ng/ml)** | 0.21030 | < 0.01 | < 0.01 |
| **Glucosa, (mg/dl)** | 0.19345 | < 0.01 | < 0.01 |
| **Creatinina, (mg/dl)** | 0.24069 | < 0.01 | < 0.01 |
| **BUN, (mg/dl)** | 0.18886 | < 0.01 | < 0.01 |
| **25 (OH) Vitamina D, (ng/ml)** | 0.07108 | < 0.01 | < 0.01 |
| **Score total de severidad** | 0.04815 | < 0.05 | < 0.01 |

Definiciones: SpO2, saturación de oxígeno por oximetría de pulso; UCI: Unidad de Cuidados Intensivos; PCR: Proteína C reactiva; LDH; Lactato deshidrogenasa, BUN: nitrógeno ureico.
