# Supplementary material for: Changes in clinical characteristics and outcomes of patients hospitalized with COVID-19 during two years of the pandemic: experience in a venezuelan hospital
Source: Rev Peru Med Exp Salud Publica. 2022 Sep 30;39(3):292–301. doi: 10.17843/rpmesp.2022.393.11195 (PMC11397598; doi:10.17843/rpmesp.2022.393.11195)
Supplement: Translation

# Translation: Cambios en las características clínicas y desenlaces de pacientes hospitalizados por COVID-19 durante dos años de pandemia: experiencia en un hospital venezolano

**Authors:** María C. Arvelo, María Montes de Oca, Laura Sánchez-Traslaviña, Flor H. Pujol, Rossana C. Jaspe, Isabel C. Silva, Irene Stulin, Gabriela Blanco, Jennireth Quevedo, Nathalia Valera, Irene Papa, Santiago Bacci, Fátima de Abreu, Héctor Villarroel, Juan C. Catari, José L. Lopez, Brigitte Moran, Claudio Cárdenas, Saverio Santucci, José L. Viloria, Eleonora García, Jerry Gómez, Antonio Martinelli, Manuel Guzmán

**Affiliations:** 1 Hospital Centro Médico de Caracas, Caracas, Venezuela.; 2 Universidad Central de Venezuela (UCV), Facultad de Medicina, Caracas, Venezuela.; 3 Laboratorio de Virología Molecular, Centro de Microbiología y Biología Celular (CMBC), Instituto Venezolano de Investigaciones Científicas (IVIC), Caracas, Venezuela.

**Keywords:** COVID-19, SARS-CoV-2, Infección por SARS-CoV-2, Unidad de Cuidados Intensivos, Mortalidad Intrahospitalaria

## Abstract

**Objetivos.:**

Determinar los cambios en las características clínicas y desenlaces intrahospitalarios de los pacientes hospitalizados por COVID-19 en un hospital privado de Caracas durante dos años de pandemia.

**Materiales y métodos.:**

Estudio retrospectivo, observacional, de pacientes hospitalizados por COVID-19. Se investigó la correspondencia entre las olas de ingresos hospitalarios con las variantes circulantes del SARS-CoV-2 en la población general del Distrito Capital y estado Miranda.

**Resultados.:**

Se incluyeron 1025 pacientes (569 hombres y 456 mujeres), con edad promedio de 62,9 DE: 16,2 años. Cuatro olas de ingresos hospitalarios fueron identificadas: primera (marzo-noviembre 2020) 150/1025 (14,6%) casos; segunda (diciembre-2020 a mayo-2021) 415/1025 (40,5%) casos; tercera (junio-diciembre 2021) 344/1025 (33,6%) casos; cuarta (enero-febrero 2022) 116/1025 (11,3%) casos. La edad promedio fue mayor en la cuarta ola (primera 64,0±15,7, segunda 61,4±15,8, tercera 62,1±16,5, y cuarta ola 68,5±16,4), mientras que la proporción de pacientes masculinos (primera 66,7%, segunda 58,8%, tercera 50,3%, y cuarta 44,8%), los pacientes con enfermedad grave-crítica (primera 65,3%, segunda 57%, tercera 51,7% y cuarta 44,8%), la estadía intrahospitalaria (primera 9,1±6,0, segunda 9,0±7,3, tercera 8,8±7,7, y cuarta 6,9±5,0 días), los ingresos a la UCI (primera 23,3%, segunda 15,7%, tercera 14,0%, y cuarta 11,2%; p=0,027) y la mortalidad (primera 21.8%, segunda 10,7%, tercera 9,1%, y cuarta 7,1%; p<0,001) disminuyeron progresivamente con el tiempo.

**Conclusiones.:**

Los resultados muestran menor frecuencia de casos severos y mejoría de los desenlaces intrahospitalarios en dos años de pandemia. Es probable que los cambios en las variantes circulantes, las mejoras del manejo de la enfermedad y la vacunación hayan influido sobre estos resultados.

## INTRODUCCIÓN

La enfermedad por coronavirus 2019 (COVID-19) es la primera pandemia en más de 100 años. En Venezuela, según datos del CENDES-COVID-19, hasta la misma fecha se registraron 515 126 casos acumulados (81 829 casos en Distrito Capital y 71 781 en el estado Miranda) y 5636 muertes (1074 decesos en Distrito Capital y 624 en el estado Miranda) ^1^. 

Se han descrito más de 2000 linajes del SARS-CoV-2, de los cuales algunos han sido denominados por la Organización Mundial de la Salud (OMS) como variantes de interés (VOI), o de preocupación (VOC), dado su impacto en la salud pública. La OMS identificó cinco VOC (alfa, beta, gamma, delta y ómicron) desde el comienzo de la pandemia y varias VOI, entre las cuales lambda y mu, que se identificaron en América Latina. Para finales de marzo de 2022 en el mundo predominaba la VOC ómicron ^2^. 

Desde el inicio de la pandemia se han implementado diferentes estrategias para reducir la transmisión del virus y su impacto en la salud. Las medidas restrictivas, como el uso de mascarillas, el distanciamiento social y los cierres masivos, dieron como resultado una reducción en el riesgo de infección ^3^ ^-^ ^5^. En el ámbito hospitalario, se han implementado varias intervenciones terapéuticas, como el uso de antivirales, antiinflamatorios, la profilaxis de trombosis y la oxigenoterapia. Finalmente, el desarrollo de vacunas desde el 2021 ha conducido a una reducción de casos de COVID-19 ^6^ ^-^ ^8^. 

Varios estudios han evaluado las características clínicas y desenlaces de los pacientes hospitalizados por COVID-19 en diferentes periodos de la pandemia ^9^ ^-^ ^19^. Aunque en algunos estudios el número de pacientes hospitalizados fue relativamente similar ^9^ ^,^ ^11^ ^,^ ^13^ ^,^ ^14^ ^,^ ^18^ entre las oleadas, la gravedad de la enfermedad, los ingresos en las unidades de cuidados intensivos (UCI) y la mortalidad tienden a disminuir con el tiempo ^9^ ^-^ ^19^. 

Ningún estudio del que tengamos conocimiento ha analizado las tendencias temporales locales de las características clínicas, la gravedad, el curso intrahospitalario y mortalidad de los pacientes hospitalizados por COVID-19 en Venezuela. El conocimiento de esta información puede ayudar a comprender mejor la carga de COVID-19 en el sistema hospitalario de nuestra localidad y contribuir a la definición de políticas públicas a nivel regional. Para abordar esta brecha se diseñó este estudio en pacientes hospitalizados por COVID-19 en el hospital privado Centro Médico de Caracas (CMC) con el objetivo de determinar los cambios en el perfil de los pacientes y sus desenlaces intrahospitalarios durante dos años de pandemia.

**MENSAJE CLAVE**

Motivación para realizar el estudio: ningún estudio ha analizado localmente las tendencias temporales de las características de los pacientes hospitalizados por COVID-19, su curso intrahospitalario y la mortalidad.

Principales hallazgos: 1025 pacientes se ingresaron en un hospital de Caracas entre marzo del 2020 y febrero del 2022 (14,6% en la primera ola, 40,5% en la segunda, 33,6% en la tercera y 11,3% en la cuarta). El porcentaje de pacientes masculinos, con enfermedad grave-crítica, la estadía intrahospitalaria, los ingresos a UCI y la mortalidad disminuyeron con el tiempo.

Implicancias en salud pública: las mejorías probablemente están asociadas con las variantes circulantes del SARS-CoV-2, las mejoras en el manejo clínico y el programa de vacunación.

## MATERIALES Y MÉTODOS

Se realizó un estudio de cohorte retrospectiva (revisión de historias clínicas) de pacientes internados con diagnóstico de COVID-19 en el hospital CMC, entre el 1 de marzo de 2020 y 28 de febrero de 2022. El hospital CMC es un hospital privado en el área metropolitana de Caracas que cuenta con todos los recursos médicos y asistenciales de un hospital privado Tipo A, según las Norma COVENIN 2339:87 ^20^. 

Según el número de casos hospitalizados en el CMC se identificaron cuatro olas durante los dos años de pandemia. La primera ola se presentó entre marzo y noviembre del 2020 (9 meses), la segunda ola se registró entre diciembre del 2020 y mayo del 2021 (6 meses), la tercera ola se registró entre junio y diciembre del 2021 (7 meses) y la cuarta y última ola fue entre enero y febrero del 2022 (2 meses).

Se usó el formulario de informe de caso (versión CORE en español del 23 de abril de 2020) desarrollado por el estudio ISARIC y la OMS ^21^ para recopilar los datos de los pacientes. Los datos recopilados fueron edad (años), grupo estadio, sexo, prueba diagnóstica para COVID-19 (antígeno-SARS-CoV-2, rRT-PCR, y IgM-IgG), números de días de síntomas previo ingreso, saturación de oxígeno de ingreso a aire ambiente (SpO2 %), gravedad de la enfermedad (leve, moderada, grave-crítica, tratamientos (antivirales, antibióticos y antiinflamatorios), días de hospitalización, días en UCI, requerimiento de ventilación mecánica invasiva, pruebas de laboratorio y tomografías de tórax (TC) de ingreso.

### Procedimientos diagnósticos de COVID-19

El diagnóstico de COVID-19 se basó en la detección de antígeno para SARS-CoV-2 o el resultado de la prueba reacción en cadena de la polimerasa por transcripción inversa en tiempo real (rRT-PCR) para SARS-CoV-2. Se tomaron las muestras de las vías respiratorias (hisopados nasofaríngeos) según las directrices de la OMS ^22^.

### Criterios de hospitalización y clasificación de la gravedad de COVID-19

El criterio más común de ingreso hospitalario fue la hipoxemia al aire ambiente y/o presencia de infiltrados pulmonares. Se reservó el ingreso de pacientes a la UCI para aquellos con insuficiencia respiratoria aguda grave que requerían oxigenoterapia con cánula nasal de alto flujo o ventilación mecánica invasiva (VMI). La gravedad de la enfermedad se estableció según los criterios del National Institute of Health ^23^.

### Análisis semicuantitativo de la TC de tórax

La afectación pulmonar y definiciones de los términos radiológicos (vidrio esmerilado, consolidado, patrón de empedrado) se basaron en el glosario de la Sociedad Fleischner ^24^. La puntuación total de gravedad de la TC de tórax de ingreso fue calculada según los siguientes criterios de Pan *et al*. ^25^: 0 (sin compromiso), 1 (compromiso <5%), 2 (compromiso de 5 a 25%), 3 (compromiso de 26 a 50%), 4 (compromiso de 51% a 75%), 5 (compromiso >75%). La puntuación total fue la suma de la afectación de cada lóbulo (rango entre 0 a 25).

Para distribuir los pacientes en cuartiles de gravedad de la puntuación total en la TC de tórax se usaron los puntos de cortes del primer, segundo y tercer cuartiles introduciendo la puntuación de todos los pacientes en una calculadora estadística de cuartiles. Los puntos de cortes para los cuartiles fueron: Q1 (puntuación entre 0-8 puntos), Q2 (puntuación entre 9-12 puntos), Q3 (puntuación entre 13-16 puntos) y Q4 (puntuación entre 17-25 puntos) ^(26)^. Cada paciente fue asignado en el cuartil correspondiente (ejemplo, una puntuación en la TC de 14 puntos ubica al paciente en el Q3).

### Análisis genómicos de las variantes SARS-CoV-2

La circulación de variantes del SARS-CoV-2 se realizó mediante la identificación de estas en muestras de la población general del Distrito Capital y estado Miranda, basado en secuenciación de un fragmento pequeño del gen de superficie del genoma viral, con confirmación de la identificación de variantes mediante secuenciación del genoma completo ^27^ ^-^ ^30^. 

Durante la primera ola no se espera que haya circulado ninguna variante y la mayoría de los linajes pertenecieron probablemente al grupo B, virus que poseen la mutación D614G en la proteína de la espiga ^27^. La segunda ola se caracterizó por la circulación mayoritaria de la variante gamma, junto con otros linajes no variantes, en enero y febrero del 2021, que fueron totalmente desplazados por la VOC gamma desde marzo del 2021 (>90%) ^28^. La tercera ola se caracterizó por una mayor diversidad de circulación de variantes, tanto VOI como VOC, manteniéndose en junio del 2021 el predominio de la VOC gamma, en agosto del 2021 una frecuencia similar de circulación de las VOC gamma y delta y de la VOI mu en menor proporción, para luego a partir de septiembre del 2021 el predominio de delta hasta final de año. La cuarta ola se caracterizó por la circulación casi exclusiva de la VOC ómicron ^30^.

### Análisis estadístico

La estadística descriptiva muestra las variables categóricas como frecuencia en número y porcentaje, mientras que las cuantitativas se muestran como mediana y rango intercuartílico (Q75-Q25). La comparación de las variables cuantitativas entre los pacientes de las cuatro olas se realizó usando la prueba no paramétrica de Kruskal Wallis por no mostrar las variables una distribución normal (Tabla suplementaria S1) y para las variables cualitativas se usó la prueba chi cuadrado de Pearson. Se consideró un valor de p de 0,05 o menos como estadísticamente significativo. Se utilizó el programa STATISTICA Versión 10 (StatSoft) para los análisis.

### Aspectos éticos

El estudio fue aprobado por la Comisión de Bioética del C.A. Centro Médico de Caracas. Por tratarse de un estudio retrospectivo de revisión de historias clínicas, el grupo de investigadores guardó la confidencialidad de los pacientes. 

## RESULTADOS

Un total de 1025 pacientes adultos (569 hombres y 456 mujeres) ingresaron con diagnóstico de COVID-19 entre el 1 de marzo del 2020 y el 28 de febrero del 2022. La edad promedio fue de 62,9 DE: 16,2 años. Dieciséis pacientes fueron trasladados a otras instituciones y un paciente permanecía hospitalizado para el cierre del estudio, por lo que los detalles de mortalidad se analizaron en 1008 pacientes. 

La Figura 1 muestra las cuatro olas registradas según el número de pacientes hospitalizados por COVID-19 por mes de ingreso y el número de casos por mes registrados por el CENDES en la población general de los estados Distrito Capital y Miranda ^1^. En ambas poblaciones se evidenciaron cuatro olas que coinciden temporalmente. En los pacientes hospitalizados se registraron 150/1025 (14,6%) casos en la primera ola (pico en agosto; 59 casos), en la segunda ola 415/1025 (40,5%) casos (pico en marzo 2021; 140 casos), en la tercera ola 344/1025 (33,6%) casos (pico en septiembre; 90 casos), y en la cuarta ola (de ascenso rápido y duración corta) 116/1025 (11,3%) casos (pico en enero; 99 casos). Similares características se observaron en la curva de casos de la población general.

**Figura 1:** Número de casos detectados con COVID-19 en la población general (Distrito Capital y Estado Miranda) y número de pacientes hospitalizados por COVID-19 según mes de ingreso en el Centro Médico de Caracas.

Las variantes circulantes durante cada ola en el Distrito Capital y el estado Miranda (entidades de donde proviene la mayoría de los pacientes hospitalizados) se muestran en la Figura 1. Las características clínicas, gravedad de la enfermedad, estadía intrahospitalaria, medición usada del total de pacientes y en cada ola se muestran en la Tabla 1. La prueba predominante para el diagnóstico en las primera y segunda olas (100 y 67%, respectivamente) fue la rRT-PCR, mientras que la detección del antígeno (SARS-CoV-2) lo fue en la tercera y cuarta (66 y 60%, respectivamente). Los pacientes de la cuarta ola mostraron mayor edad. La proporción de pacientes masculinos, con enfermedad grave-crítica, los días de síntomas previos al ingreso y estadía intrahospitalaria disminuyeron significativamente con el avance de la pandemia, mostrando los pacientes de las primera y segunda olas predominio de hombres (67 y 59%, respectivamente) y enfermedad grave-crítica (65 y 57%, respectivamente), mientras que los de la cuarta ola tenían mayor proporción de mujeres (55%) y menor proporción de pacientes graves-críticos (45%). La SpO_2_ al ingreso fue significativamente menor en los pacientes de la primera ola (p = 0,030). Un total de 87% de los pacientes recibieron remdesivir, 34,1% antibióticos, 70,3% esteroides sistémicos y 3,6% tocilizumab. Solo 41,3% de los pacientes de la primera ola recibieron remdesivir comparado con más del 90% de los de la segunda, tercera y cuarta olas. El uso de antibióticos fue superior al 55% en la primera ola comparado con 30% aproximadamente en los de otras olas. Se observó disminución progresiva del uso de esteroides sistémicos con el avance de la pandemia y mayor uso de tocilizumab en las tercera y cuarta olas.

**Tabla 1:** Características clínicas, estadía hospitalaria, uso de medicación y ventilación mecánica invasiva de pacientes hospitalizados por COVID-19 y en las cuatro olas

Variables	Total n=1025 n (%)	Ola 1 n=150 n (%)	Ola 2 n=415 n (%)	Ola 3 n=344 n (%)	Ola 4 n=116 n (%)	Valor de p
Edad	63,5 (23,0)	66,0 (22,0)	62,0 (22,0)	62,0 (25,0)	72,5 (21,5)	<0,001^a^
Grupo etario, (%)						
≤30 años	28/1023 (2,8)	3/150 (2,0)	11/415 (2,7)	11/344 (3,2)	3/116 (2,6)	0,043^b^
31-50 años	210/1023 (20,5)	30/150 (20,0)	93/415 (22,4)	75/344 (21,8)	12/116 (10,3)	
51-65 años	297/1023 (29,0)	36/150 (24,0)	128/415 (30,8)	100/344 (29,1)	30/116 (25,9)	
>65 años	488/1023 (47,7)	81/150 (54,0)	183/415 (44,1)	158/344 (45,9)	71/116 (61,2)	
Sexo, (%)						
Masculino	569/1025 (55,5)	100/150 (66,7)	244/415 (58,8)	173/344 (50,3)	52/116 (44,8)	<0,001^b^
Femenino	456/1025 (44,5)	50/150 (33,3)	171/415 (41,2)	171/344 (49,7)	64/116 (55,2)	
Diagnóstico COVID-19, (%)						
Antígeno (SARS-CoV-2)	415/985 (42,1)	0/143 (0)	126/392 (32,1)	219/334 (65,6)	70/116 (60,3)	<0,001^b^
rRT-PCR	567/985 (57,6)	143/143 (100)	264/392 (67,4)	114/334 (34,1)	46/116 (39,7)	
IgM-IgG	3/985 (0,3)	0/143 (0,0)	2/392 (0,5)	1/334 (0,3)	0/116 (0)	
Días síntomas previo ingreso	7,0 (5,0)	7,0 (5,0)	7,0 (5,0)	7,0 (5,0)	6,0 (4,0)	<0,001^a^
Estadios días síntomas, (%)						
Temprano (0-5 días)	360/975 (36,9)	52/149 (34,9)	124/384 (32,3)	129/328 (39,3)	55/114 (48,2)	0,032^b^
Progresivo (6-8 días)	331/975 (33,9)	46/149 (30,9)	135/384 (35,2)	113/328 (34,4)	37/114 (32,5)	
Pico (9-13 días)	188/975 (19,3)	31/149 (20,8)	89/384 (23,2)	53/328 (16,2)	15/114 (13,2)	
Tardío (≥14 días)	96/975 (9,9)	20/149 (13,4)	36/384 (9,4)	33/328 (10,1)	7/114 (6.1)	
SpO_2_ % ingreso, (FiO_2_ 0,21)	94,0 (6,0)	93,0 (7,0)	94,0 (6,0)	95,0 (6,0)	95,0 (6,0)	0,030^a^
Gravedad COVID-19, (%)						
Leve	68/1024 (6,6)	7/150 (4,7)	12/414 (2,9)	22/344 (6,4)	27/116 (23,3)	
Moderada	392/1024 (38,3)	45/150 (30,0)	166/414 (40,1)	144/344 (41,9)	37/116 (31,9)	<0,001^b^
Grave-Critica	564/1024 (55,1)	98/150 (65,3)	236/414 (57,0)	178/344 (51,7)	52/116 (44,8)	
Remdesivir, (%)	896/1025 (87,4)	62/150 (41,3)	405/415 (97,6)	324/344 (94,2)	105/116 (90,5)	0,027^b^
Antibióticos, (%)	349/1024 (34,1)	85/150 (56,7)	125/415 (30,1)	104/344 (30,2)	35/115 (30,4)	<0,001^b^
Esteroides sistémicos, (%)	721/1025 (70,3)	132/150 (88)	312/415 (75,2)	211/344 (61,3)	66/116 (56,9)	<0,001^b^
Tocilizumab, (%)	37/1025 (3,6)	0/150 (0)	2/415 (0,5)	26/344 (7,6)	9/116 (7,8)	<0,001^b^
Días hospitalización	6,0 (4,0)	8,0 (7,0)	7,0 (3,0)	6,0 (3,0)	6,0 (3,0)	<0,001^a^
Días en UCI	10,0 (10,0)	9,5 (9,5)	10,0 (11,0)	12,5 (15,5)	6,0 (4,0)	0,036^a^
VMI, (%)	150/1024 (14,6)	26/150 (17,3)	47/344 (11,3)	40/344 (11,6)	12/116 (10,3)	0,217^b^

rRT-PCR: prueba reacción en cadena de la polimerasa por transcripción inversa en tiempo real; IgM-IgG: inmunoglobulina G-M; SpO_2_: saturación de oxígeno por oximetría de pulso; UCI: unidad de cuidados intensivos; VMI: ventilación mecánica invasiva, (%).

Los datos se muestran como n (%) o mediana (rango intercuartílico).

a Prueba de Kruskal Wallis, ^b ^Prueba de chi cuadrado de Pearson

La Tabla 2 muestra los valores de algunas pruebas de laboratorio al ingreso del total de pacientes y de las olas. Los pacientes de la primera ola mostraron valores significativamente más elevados de recuento de leucocitos, neutrófilos, plaquetas, y BUN, mientras que los de la cuarta ola presentaron niveles más bajos de hemoglobina, hematocrito, ferritina y vitamina D. No se observaron diferencias en los valores de linfocitos, glicemia, PCR y HDL.

**Tabla 2:** Resultados de laboratorio de ingreso en el total de pacientes hospitalizados por la COVID-19 y en las cuatro olas.

Variables	Total n=1025	Ola 1 n=150	Ola 2 n=415	Ola 3 n=344	Ola 4 n=116	Valor de p ^a^
Hemoglobina, gr/dL	14,1 (2,3)	14,4 (2,2)	14,5 (2,2)	13,5 (2,0)	13,2 (1,9)	<0,001
Hematocrito, (%)	42,6 (7,0)	43,3 (6,7)	44,9 (6,6)	41,0 (6,5)	40,4 (5,7)	<0,001
Leucocitos, (x10^9^/L)	6700 (4400)	7300 (4900)	6600 (4450)	6300 (3800)	7300 (4600)	0,001
Neutrófilos, (x10^9^/L)	4900 (4200)	5600 (4151)	4900 (4200)	4600 (3800)	5000 (4450)	0,005
Linfocitos, (x10^9^/L)	900 (700)	981,5 (700)	900 (700)	900 (700)	1000 (650)	0,168
Plaquetas, (x10^9^/L)	203,0 (106,0)	224,0 (132,0)	201,0 (97,5)	193,0 (97,0)	216,0 (93,0)	0,004
PCR, (mg/dL)	5,05 (8,4)	5,6 (6,0)	4,8 (9,3)	4,8 (8,8)	4,8 (9,9)	0,507
LDH basal, (u/L)	273,0 (137,5)	286,5 (165,5)	276,5 (142,0)	271,0 (137,0)	258,0 (95,0)	0,223
Dímero D, (µg/L)	0,83 (1,07)	1,02 (2,01)	0,68 (0,73)	0,80 (0,88)	1,49 (2,12)	<0,001
Ferritina, (ng/mL)	395,1 (464,8)	577,1 (483,7)	384,4 (439,5)	443,1 (581,0)	217,0 (303,5)	<0,001
Glucosa, (mg/dL)	108 (38)	107 (40)	106 (37)	109 (35)	113 (46)	0,125
Creatinina, (mg/dL)	1,0 (0,33)	0,99 (0,29)	0,97 (0,33)	1,02 (0,36)	1,01 (0,39)	0,018
BUN, (mg/dL)	15,0 (8,0)	17,4 (13,6)	15,0 (8,2)	14,0 (6,0)	16,0 (7,0)	<0,001
25 (OH) Vitamina D, (ng/mL)	25,8 (16,9)	24,7 (14,7)	26,2 (16,0)	29,1 (19,5)	16,6 (16,9)	<0,001

PCR: proteína C reactiva; LDH; lactato deshidrogenasa, BUN: nitrógeno ureico.

Los datos se muestran como mediana (rango intercuartílico).

a Prueba de Kruskal Wallis

La gravedad en la puntuación de la TC de tórax de ingreso y la distribución de los pacientes según el cuartil de gravedad tomográfica que presentaban los pacientes al ingreso del total de pacientes y las cuatro olas se muestran en las Figuras 2A-2B. La puntuación de gravedad el porcentaje de pacientes en los cuartiles más graves (Q3-Q4) disminuyó progresivamente con el tiempo de pandemia. La mayor puntuación se observó en la primera ola y la menor en la cuarta. Un 52,6% de los pacientes de la primera ola se ubicaron en los cuartiles más graves (Q3-Q4), comparado con 31,3% de los ubicados en los cuartiles Q3-Q4 en la cuarta ola.

**Figura 2:** A. Puntuación de gravedad en la tomografía de tórax en la población total de pacientes y en las cuatro oleadas. B. Distribución de los pacientes en los cuartiles de gravedad de la tomografía de tórax en la población total y en las cuatro oleadas.

Los ingresos a la UCI y la mortalidad del total de pacientes y en las olas se muestran en la Figura 3. Un total de 161/1025 (15,7%) pacientes ingresaron a la UCI y la mortalidad general fue 11,3% (114/1008). A medida que avanzó la pandemia se observó disminución progresiva en los ingresos a la UCI (primera 23,3%, segunda 15,7%, tercera 14,0%, y cuarta 11,2%; p = 0,027) y en la mortalidad (primera 21,8%, segunda 10,7%, tercera 9,1%, y cuarta 7,1%; p < 0,001). Al comparar los ingresos a la UCI y mortalidad entre las olas solo se observó diferencia significativa entre la primera ola con la segunda, tercera y cuarta (Figura 4).

**Figura 3:** Proporción de pacientes hospitalizados por COVID-19 con ingresos en la unidad de cuidados intensivos (UCI) y mortalidad total, en las cuatro olas.

La Figura 4 muestra el esquema de vacunación contra la COVID-19 que recibieron los pacientes ingresados entre julio del 2021 y febrero del 2022. Se observó disminución progresiva de la proporción de pacientes no vacunados y aumento de aquéllos con esquema completo con el tiempo. En la tercera ola, el 39,4% de los pacientes tenían un esquema de vacunación completo comparado con 83,5% de los de la cuarta. Un total de 2/178 pacientes (1,1%) de la tercera ola y 20/100 pacientes (20%) de la cuarta ola tenían dosis de refuerzo. Del total de los 39 pacientes fallecidos en las tercera y cuarta oleadas, 16 tenían esquema de vacunación completo. Las vacunas más usadas como esquema primario fueron: Sinopharm o Sinovac con 178 casos (64,3%), Sputnik V con 88 casos (31,7%), Pfizer con nueve casos (3,2%), Moderna con un caso (0,4%) yJohnson & Johnson con un caso (0,4%).

**Figura 4:** Esquema de vacunación contra la COVID-19 de los pacientes hospitalizados entre julio y diciembre del 2021.

## DISCUSIÓN

Los principales hallazgos de este estudio sobre características clínicas y desenlaces de pacientes hospitalizados por COVID-19 en dos años de pandemia fueron: primero, se registraron diferencias en el número de casos hospitalizados durante la pandemia. En la primera ola se ingresó 14,6% del total de pacientes, mientras que en la cuarta 11,3%. La segunda y tercera ola tuvieron el mayor número de ingresos (40,5% y 33,7%, respectivamente). Un comportamiento similar se observó en el número de casos registrados en la población general. Segundo, la edad promedio fue mayor en la cuarta ola, mientras que la proporción de pacientes masculinos, la gravedad de la enfermedad, la estadía intrahospitalaria, los ingresos a la UCI y la mortalidad disminuyeron progresivamente con el tiempo. 

Varios estudios han evaluado el cambio en el número de ingresos hospitalarios por COVID-19 en las primeras oleadas de la pandemia ^9^ ^-^ ^11^ ^,^ ^13^ ^,^ ^14^ ^,^ ^17^ ^-^ ^19^. En Estados Unidos un análisis del 2020 reportó 11901 pacientes ingresados entre marzo y abril, 4116 entre mayo y junio, 2709 entre julio y agosto, y 2010 entre septiembre y noviembre ^10^. En general, los estudios europeos reportan un incremento en los ingresos hospitalarios por la COVID-19 durante dos períodos epidémicos del 2020 ^9^ ^-^ ^11^ ^,^ ^13^ ^,^ ^14^ ^,^ ^17^ ^-^ ^19^. Un estudio en España reportó 2547 pacientes hospitalizados en la primera ola (febrero y mayo del 2020) y 2673 en la segunda (junio y septiembre del 2020) ^13^. Datos de un hospital en Madrid indican 1788 ingresos (48,6%) entre marzo y junio del 2020, 926 (25,2%) entre julio y noviembre del 2020 y 962 (26,2%) entre diciembre del 2020 y abril del 2021. Similar tendencia fue reportada en Alemania e Italia ^11^ ^,^ ^17^ ^,^ ^19^. Un estudio en Brasil reportó 325 903 (48,05%) pacientes hospitalizados en la primera oleada (febrero y noviembre del 2020) y 352 332 (51,95%) en la segunda (noviembre del 2020 y abril del 2021) ^18^. 

Aunque la tendencia del número de hospitalizaciones por la COVID-19 en el presente estudio va en línea con los estudios anteriores al mostrar incremento progresivo de los ingresos entre la primera y segunda ola, con disminución posterior en la tercera y cuarta, es importante destacar que los resultados son difíciles de comparar, ya que las oleadas entre los estudios son desiguales por ser regiones con distintas curvas epidemiológicas en las que probablemente han influido diferencias estacionales, en las variantes circulantes, las medidas restrictivas y la vacunación. Es probable que los principales factores que han influido en el presente estudio sobre el elevado número de ingresos durante las segunda y terceras olas sean la elevada trasmisibilidad y patogenicidad de las variantes circulantes (gamma y delta) y la flexibilización de las medidas restrictivas. Por otra parte, el predominio de la variante ómicron (de alta transmisibilidad y baja patogenicidad) durante la cuarta ola, más el avance en la vacunación, son probablemente los factores con mayor influencia en el comportamiento de los ingresos en ese período ^30^ ^-^ ^32^. 

Los estudios que comparan las oleadas en un año de pandemia en general muestran un predominio de pacientes del sexo masculino ^9^ ^,^ ^10^ ^,^ ^13^ ^,^ ^17^ ^-^ ^19^, aunque algunos resaltan un ligero incremento de mujeres con el tiempo ^10^. También muestran mayor edad, y peores parámetros de gravedad en las primeras oleadas ^9^ ^,^ ^10^ ^,^ ^13^ ^,^ ^17^ ^,^ ^18^. Por otro lado, los desenlaces intrahospitalarios mostraron una tendencia a mejorar con el tiempo en otras series^ (^ ^9^ ^-^ ^11^ ^,^ ^13^ ^,^ ^14^ ^,^ ^17^. Hay estudios que reportaron disminución de la estadía intrahospitalaria (22 vs. 14 días), de los ingresos a la UCI (17,1% vs. 7,2%) y la mortalidad (24,0% vs. 13,2%) entre la primera y segunda ola ^9^. Datos en Estados Unidos del 2020 indican que la mortalidad fue: 19,1% entre marzo y abril, 11,9% entre mayo y junio, 11,0% entre julio y agosto y 10,8% entre septiembre y noviembre ^10^. Un estudio en España mostró que los ingresos en UCI fueron 16% y 10%, y la mortalidad acumulada fueron 38% y 32% en la primera y segunda ola, respectivamente ^13^. Similares resultados sobre mortalidad han sido reportados en Italia ^17^. Otros autores informaron que la estadía hospitalaria (14,5 vs. 8 días) y los ingresos en UCI (31,9% vs. 13,3%) se redujeron entre las oleadas, pero no la mortalidad (14,1 vs. 11,4%) ^14^. En contraste, otros estudios no encontraron diferencias en los ingresos a UCI o la mortalidad o reportan incremento en estos desenlaces ^18^ ^,^ ^19^. 

Existe escasa información sobre las características clínicas y desenlaces intrahospitalarios de los pacientes con COVID-19 más allá del primer año de la pandemia. Estudios recientes reportan disminución de la gravedad de la enfermedad en la oleada por ómicron comparada con períodos anteriores ^32^ ^-^ ^35^. En California se observó que los pacientes hospitalizados durante el período de ómicron tenían mayor edad (66 vs 60 años), menor gravedad, menos ingreso en la UCI (16,8 % vs 23,3 %), menor necesidad de VMI (9,2 % vs 13,6 %) y menos fallecidos (4,0 % vs 8,3%) comparado con los del período delta ^34^. Parte de este efecto parece deberse a una mayor proporción de pacientes vacunados ^34^. 

Los hallazgos del presente estudio están en línea con lo reportado previamente al mostrar predominio en las olas iniciales de pacientes del sexo masculino y mejoría progresiva de los parámetros clínicos y tomográficos de la enfermedad, así como reducción de la estadía hospitalaria, los ingresos a UCI y la mortalidad. Igualmente muestran durante el período de predominio de la variante ómicron incremento en la edad, mayor proporción de mujeres, menor gravedad en la TC de tórax, disminución de la estadía hospitalaria, los ingresos en la UCI, y la mortalidad.

Múltiples factores pueden ayudar a justificar los hallazgos del presente estudio. Una explicación para las diferencias en la mortalidad podría ser las mejoras en el manejo clínico de los pacientes incluyendo la farmacoterapia como el uso de anticoagulantes, y esteroides sistémicos. Otros cambios en pacientes más graves como el uso más racional de antibióticos o tocilizumab probablemente estén asociado con los resultados. La disminución en la edad (10% menor el grupo >65 años) y en la proporción de hombres (8-16% menor) en la segunda y tercera ola respecto a la primera, también pueden haber influido en la disminución de la letalidad. Es probable que el aumento de hospitalización de grupos más jóvenes durante las segunda y tercera ola pueda estar parcialmente relacionado con la vacunación, que en un principio priorizó los grupos de edad avanzada. Finalmente, la disminución en la gravedad y mejoría en los desenlaces también podrían explicarse parcialmente por los cambios de las variantes circulantes ^27^ ^-^ ^30^. Durante la primera ola no circuló ninguna variante, la segunda se caracterizó por la circulación mayoritaria de la variante gamma, la tercera por las variantes gamma, delta y mu y la cuarta por ómicron ^27^ ^-^ ^30^. Aunque las mutaciones en las nuevas variantes se relacionan con mayor infectividad y carga viral, la evidencia indica asociación con menor gravedad de la enfermedad. Durante el periodo de predominio de ómicron, además de la menor patogenicidad de esta variante, es probable que el desarrollo de la vacunación (esquemas completos > 80% de los pacientes), incluso con dosis de refuerzo, esté asociado con la menor gravedad de los casos, la disminución de ingreso a UCI y mortalidad ^34^. Un hallazgo del presente estudio consistente con la menor patogenicidad de ómicron y la protección de las vacunas serían los mejores desenlaces (ingresos a UCI y mortalidad) observados en este período a pesar de la mayor edad de los pacientes (61% ≥ 65 años).

El presente estudio tiene algunas limitaciones. Primero, es un estudio observacional retrospectivo, por lo que existen factores de confusión no medidos que carecen del nivel de detalles necesarios para un análisis extenso sobre los desenlaces. No obstante, estos resultados concuerdan con los reportados en otras series y representan el primer análisis en una muestra grande de pacientes hospitalizados por la COVID-19 en nuestra población durante dos años de pandemia, con información sobre las variantes circulantes localmente. Segundo, nuestro centro es un hospital privado en Caracas, lo que puede influir en las características de los pacientes y limitar la generalización de los resultados a otros entornos. Tercero, el estudio solo analizó pacientes hospitalizados quienes, en general, tenían enfermedad moderada a grave-crítica, lo que limita la generalización de los hallazgos a pacientes menos graves tratados de forma ambulatoria. Cuarto, no se analizó la variante infectante en cada uno de los pacientes hospitalizados, sino que se evaluó la correspondencia de cada ola con la diversidad viral circulante en cada período.

En conclusión, los resultados del presente estudio muestran cambios con el tiempo en las características clínicas de los pacientes hospitalizados por la COVID-19 y mejoría progresiva en la gravedad de la enfermedad y los desenlaces intrahospitalarios. Es probable que los cambios en las variantes circulantes, la mejora en el manejo de la enfermedad y la vacunación hayan influido sobre los resultados. Los hallazgos pueden ayudar a comprender mejor la evolución de los pacientes con la COVID-19 que requieren manejo intrahospitalario en nuestro entorno y contribuir a la definición de las políticas sanitarias.
